# Experimental Study on the Bending and Shear Behaviors of Chinese Paulownia Wood at Elevated Temperatures

**DOI:** 10.3390/polym14245545

**Published:** 2022-12-18

**Authors:** Lingfeng Zhang, Biao Xu, Zhongjie Fang, Chen Li, Xijun Kong, Qianyi Li, Kai Guo

**Affiliations:** 1 College of Civil Science and Engineering, Yangzhou University, Yangzhou 225127, China; 2 College of Civil Engineering and Architecture, Yangzhou Polytechnic Institute, Yangzhou 225127, China; 3 Department of Architecture, Built Environment and Construction Engineering, Politecnico di Milano, Piazza Leonardo da Vinci 32, 20133 Milano, Italy

**Keywords:** Paulownia wood, elevated temperatures, fire, shear behavior, bending behavior

## Abstract

Chinese Paulownia wood has been extensively used in the construction of timber buildings and lightweight sandwich structures. However, the bending and shear behaviors at elevated temperatures were not well understood. A total of 162 specimens were tested to investigate the bending, tangential shear, and radial shear performances of Chinese Paulownia wood under temperatures from 20 to 220 °C. It was found that the bending specimens exhibited ductile failure due to the progressive damage after reaching the peak load, while the tangential and radial shear specimens exhibited brittle shear failure along the shear plane. The elevated temperatures had limited effects on the failure modes. Under the same temperature, the retention rate of the modulus of elasticity is significantly higher than that of the modulus of rupture. Moreover, the bending strength, tangential shear strength, and radial shear strength generally and nonlinearly decreased with the increasing temperature. The EN 1995-1-2 design curve for the shear strength of wood at elevated temperatures is conservative for both the tangential and radial shear specimens. However, the design curve may not be adopted to estimate the tangential shear strength at temperatures higher than 220 °C.

## 1. Introduction

In China, timber structures have a long history. In ancient times, wood material was commonly used as the building material in the construction of temples, palaces, pavilions, and houses. There are several existing scenic spots made of timber structures, i.e., the Forbidden City (which has the largest ancient timber building groups) and the Yingxian wooden tower (the tallest ancient wooden tower). With the development of technologies in processing, manufacturing, design, and construction, wood material is being increasingly applied in the construction of modern timber building structures. Compared with traditional construction materials, such as concrete and steel, wood has the advantages of being lightweight, renewable, and prefabricated, with high specific strength, good thermal insulation, and fast installation [1,2]. However, for timber structures, one of the most significant problems is the charring and combustion behavior when the timber members are subjected to elevated and high temperatures [3,4]. Several studies have been conducted to investigate the fire performances of timber members [4,5,6,7,8]. Schaffer et al. [6] developed a model considering the temperature-dependent lumber strength, stiffness, finger-joint strength, and length of laminating lumber in the finger joints to predict the fire resistance (time to failure) of the glued-laminated beams. This model was validated by the fire experiments of 21 glued-laminated beams. The influence of structural adhesives on the fire resistance of glued-laminated beams was reported by Klippel and Frangi [9]. By conducting large-scale fire experiments, it was found that the increase of the thickness of the zero-strength layer, depending on the structural adhesive, was not required. Apart from the glued-laminated beams, the fire resistance of the cross-laminated timber (CLT) floors [5,7,10,11,12], CLT walls [7,12,13], and CLT floor-to-wall connections [14], was investigated in standard fire conditions [5,11,13] and natural fire conditions [15]. It was found that the fire resistance of the CLT elements mainly depended on the adhesive types, the number of layers (thicknesses), fire curves, and loading ratios. The reduced cross-section method (RCSM) was frequently adopted in the fire design of the timber structural elements to estimate the mechanical behavior and the fire resistance [4].

When subjected to high temperatures, wood will be decomposed into char and gases. As a result, the mechanical properties will monotonously decrease with the increase in temperature. However, at elevated temperatures, the previous studies showed that the mechanical properties exhibited nonlinear characteristics with the increase in temperature. Yang [16] reported the bending behavior of the larch and Douglas fir wood at 20–280 °C. The study found that the bending strength of the larch wood decreased rapidly from 20 to 160 °C, gradually flattened from 160 to 220 °C, and then decreased from 250 to 300 °C. For Douglas fir wood, the bending strength at 100 °C was about 60% of that at room temperature. However, the bending strength exhibited a significant increase from 100 °C to 160 °C followed by a significant reduction from 190 to 280 °C. Zhong et al. [17] carried out the three-point bending tests on the larch wood at and after elevated temperatures. The results showed that with the increase in temperature, the bending strength and elastic modulus generally decreased, while the post-heated (after exposure to elevated temperatures) specimens showed a nonlinear reduction in the bending strength. Korkut et al. [18] found that the degradation of the bending strength of the Rowan wood is higher than those of the tensile strength parallel to the grain and the compressive strength parallel to the grain. The influences of elevated temperatures on the mechanical behaviors of beech and lime wood were reported by Tajvidi et al. [19]. It was found that the modulus of elasticity (MOE) and the modulus of rupture (MOR) significantly decreased with the increase in temperature. The cylindrical shape specimens were adopted by Dhima et al. [20] to study the effects of elevated temperatures on the shear behavior of the glulam wood. It was found that the reduction factors given by EN 1995-1-2 [21] were conservative compared to the experimental result. Garrido et al. [22] reported the influence of elevated temperatures on the shear response of end-grain balsa wood. It was found that the shear strength and modulus exhibited linear and comparable reductions with the increase in temperature, and the retention rate (compared to the room temperature properties) at 240 °C was less than 0.2.

The timber beams and panels made of Chinese Paulownia wood (Paulowniaceae Nakai) have been extensively used in the construction of timber buildings and lightweight sandwich structures. Although the mechanical behavior of Chinese Paulownia wood under room temperature is well known, the mechanical properties of the wood at elevated temperatures are yet to be understood. Moreover, the bending and shear ruptures are the dominant failure modes for the timber beams and panels subjected to transverse loads. Therefore, this study investigates the effects of elevated temperatures on the bending and shear behaviors of Chinese Paulownia wood. The failure modes, strengths, modulus, and deformation capacities at elevated temperatures are presented and discussed.

## 2. Experimental Program

### 2.1. Material and Specimens

**Figure 1:**
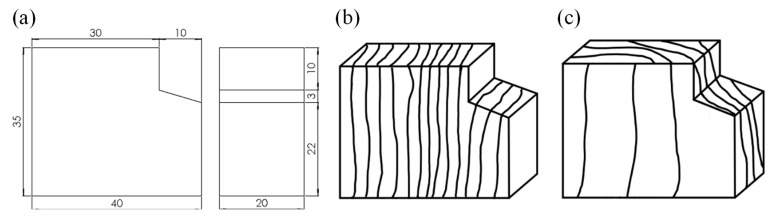
Diagram of shear specimens: (**a**) dimension (in mm); (**b**) tangential shear; and (**c**) radial shear.

All defect-free specimens (without defects and knots) used in this study were cut from the same log of Chinese fast-grown Paulownia. All specimens were first placed in the conditioned room (20 ± 2 °C, 65 ± 3% relative humidity). According to GB/T 1931-2009 [23] and GB/T 1933-2009 [24], the air-dry density of the wood samples with moisture content of 10.9 ± 0.013% was 353 ± 26.2 kg/m^3^. According to GB/T 1936.1-2009 [25], the size of the bending specimens was 300 mm × 20 mm × 20 mm and the longitudinal direction was parallel to the grain. For the bending specimen, the width is along the radial direction while the thickness is along the tangential direction. As a result, the load was applied to the tangential plane nearest to the pith. The dimension of the shear specimen is shown in Figure 1a. According to GB/T 1937-2009 [26], the three-dimensional (3D) appearances of the tangential shear specimen and the radial shear specimen are shown in Figure 1b,c, respectively.

The specimens were tested under elevated temperatures of 20, 60, 100, 120, 140, 160, 180, 200, and 220 °C. Since the mechanical properties of Paulownia wood exhibited considerable deviations, six repeated specimens were tested for each temperature group. A total of 54 bending specimens, 54 tangential shear specimens, and 54 radial shear specimens were fabricated and tested in this study.

### 2.2. Experimental Instruments and Set-Up

**Figure 2:**
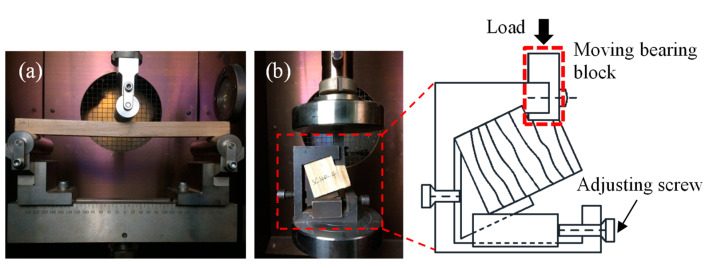
Experimental set-up: (**a**) three-point bending; (**b**) shear.

As shown in Figure 2a, the three-point bending configuration was adopted to study the bending performance of the Paulownia wood. The span of the bending specimen was 240 mm. Before the test, a reference specimen with a thermocouple in the center was placed on the support in the chamber to measure the time required to reach the desired temperature. Subsequently, the specimens to be tested were placed in the heating chamber and heated to the desired temperature (according to the required time, see Table 1); the time span between reaching the desired temperature and the start of the test was 10 min. The bending specimens were tested at a cross-head speed of 5 mm/min up to the failure. It should be noted that the specimens were still in the heating chamber during the loading process. The instrument and the fixture for the bending and the shear specimens are shown in Figure 2b. The shear specimen was first placed in the heating chamber and fixed in the shear fixture by adjusting the two screws. After reaching the desired temperature at 10 min, the load was vertically applied through the bearing block on the top surface of the specimen using the displacement control with a speed of 2 mm/min. During the tests, the temperature of the heating chamber, load, and displacement were recorded by the testing systems. It should be noted that five wood cubes with a length of 20 mm and the test specimen were simultaneously placed in the chamber. After the mechanical test, the wood cubes were weighted to measure the temperature-dependent moisture content.

### 2.3. Calculation of Bending and Shear Properties

According to GB/T 1936.1-2009 [25], the bending strength (modulus of rupture, MOR) is calculated as Equation (1):

(1) $$\sigma =\frac{3Pl}{{2bh}^{2}}$$

in which *σ* is the bending strength; *P* is the peak load; *l* is the span between the two supports; *b* is the width and *h* is the depth. The modulus of elasticity, MOE, can be calculated as Equation (2) Eaccording to [27]:

(2) $$\mathrm{MOE}=\frac{23\Delta Pl^{3}}{{108bh}^{3}\delta }$$

where Δ*P* is the load difference while *δ* is the mid-span deflection difference. The tangential shear strength and the radial shear strength can be calculated according to Equation (3) in GB/T 1937-2009 [26]:

(3) $$\tau =\frac{0.96P}{bl}$$

where *τ* is the shear strength; *P* is the peak load; *b* and *l* are the width and length of the shear plane, respectively.

## 3. Experimental Results and Discussion

### 3.1. Experimental Observation and Failure Mode

**Figure 3:**
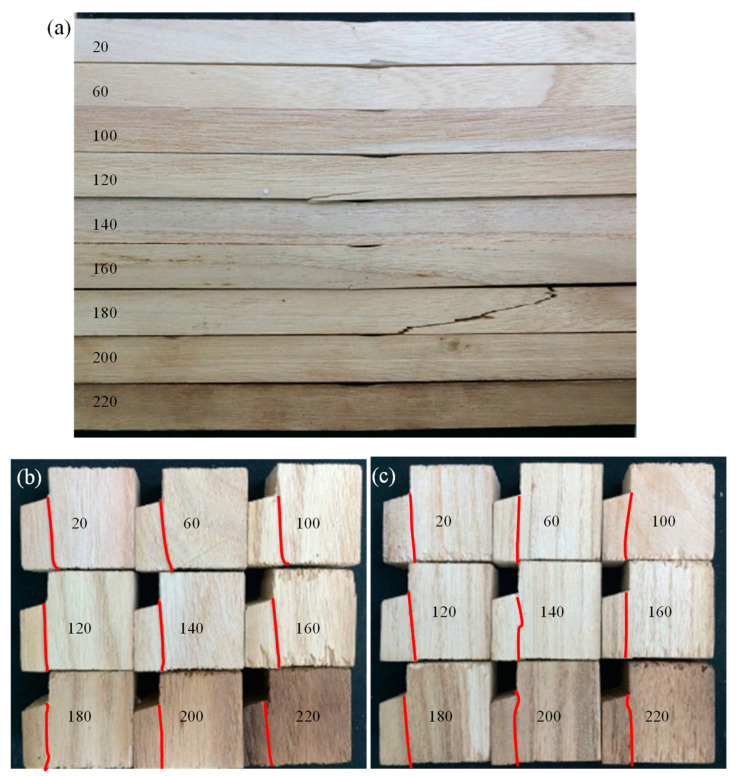
Appearances of the specimens after (**a**) bending, (**b**) tangential shear, and (**c**) radial shear failures.

**Figure 4:**
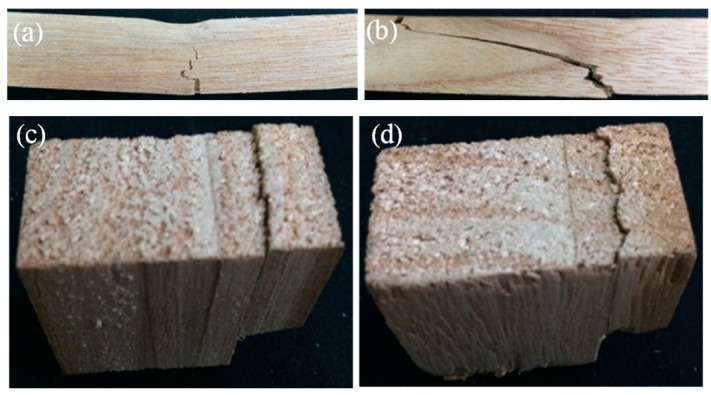
Typical failure modes: (**a**) bending crack in the bending specimen (**b**) shear crack in the bending specimen; (**c**) straight crack in the tangential shear specimen; (**d**) wave-form crack in the radial shear specimen.

**Figure 5:**
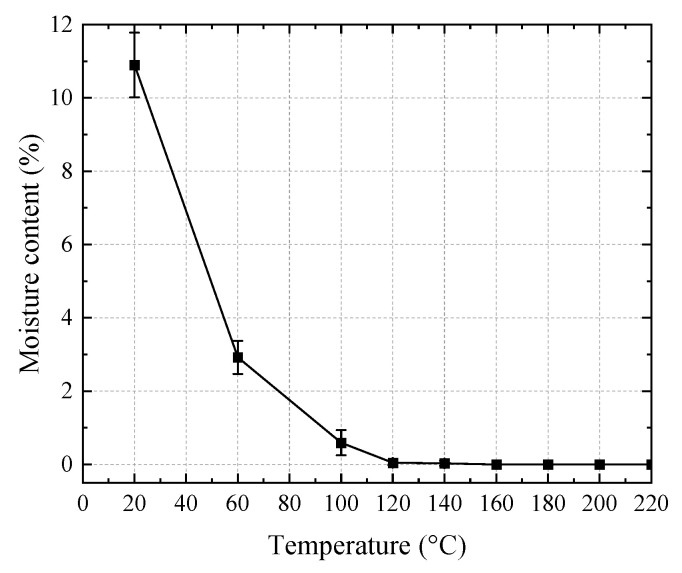
Temperature-dependent moisture content of the wood.

An overview of the bending specimens after unloading is shown in Figure 3a. The color of the specimens gradually deepened with the increase in temperature. During the tests at 200 and 220 °C, volatile gas escaped from the specimen in the chamber, indicating that significant thermal degradation occurred. Moreover, the elevated temperatures had limited effects on the failure modes. There were two typical failure modes in the bending specimens. As shown in Figure 4a, the first type is the bending failure mode: at the middle span, compressive crushing was observed at the top region while tensile failure was found at the bottom region. As shown in Figure 4b, the second type is the shear failure mode: the shear crack was obliquely extended from the bottom of one cross-section to the top of another cross-section in a few specimens. Figure 3b presents the overview of the appearances of the tangential shear specimens after unloading. A clear shear crack was formed on the side surface of the specimens. Figure 4c shows the typical crack shape on the bottom of the specimen. The shear crack is approximately straight along the tangential direction. Figure 3c and Figure 4d show the cracking shapes on the side surfaces and the bottom surfaces for radial shear specimens, respectively. Unlike the tangential shear specimen, the crack on the bottom of the radial shear specimen exhibited a waveform along the radial direction. This was due to the difference in the shear behavior between the earlywood and latewood. The temperature-dependent decrease in the moisture content is depicted in Figure 5. The moisture content dramatically reduced from 10.9% at 20 °C to 2.9% at 60 °C, then gradually decreased to approximately 0.6% at 100 °C. Moreover, the moisture content was zero (the wood is totally dried) when the temperature was above 120 °C.

### 3.2. Stress-Displacement Curves

**Figure 6:**
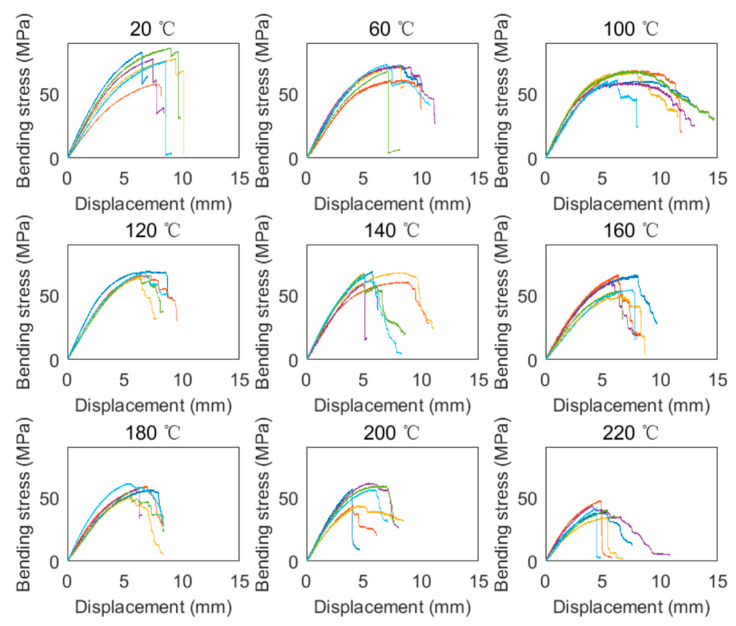
Bending stress–displacement curves at different temperatures.

The bending stress can be obtained by substituting the load into Equation (1). Figure 6 shows the bending stress versus vertical displacement curves of the specimens at different temperatures. The color in the figure denotes the index of the repeated specimen. For the specimens at 20 °C, the bending stress increased linearly and then increased nonlinearly up to the peak strength. Subsequently, most of the post-peak curves exhibited pseudo-ductile characteristics due to the progressive damage in the specimens. It should be noted that the post-peak curves of a few specimens showed brittle rupture behaviors since the bending stress suddenly and dramatically dropped. The sudden drop of the bending stress mainly came from the brittle shear failure without a progressive damage process. Moreover, since defect-free wood was adopted in the tests, the specimens in the same temperature group exhibited similar stiffness (slope of the curve) at the initial loading stage. For the specimens at elevated temperatures, similar shapes (characteristics) were found.

**Figure 7:**
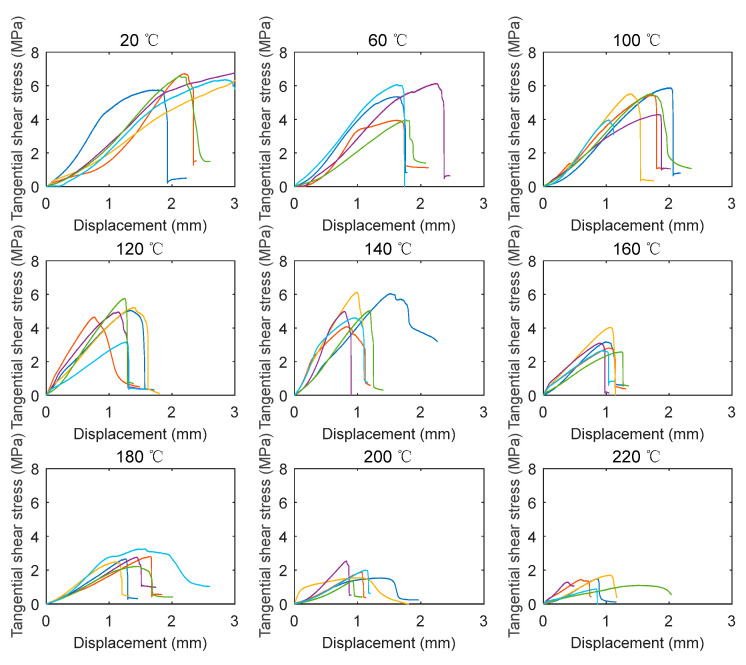
Tangential shear stress–displacement at different elevated temperatures.

The tangential shear stress and radial shear stress can be obtained by substituting the load into Equation (3). Figure 7 depicts the tangential shear stress–vertical displacement relationships of the tested specimens under different temperatures. For the specimens under 20 °C, at the initial stage, the loading steel block gradually came into contact with the specimen. As a result, the tangential shear stress increased slowly with the increase of the vertical displacement at this stage. Subsequently, the tangential shear stress increased linearly followed by the nonlinear increase up to the peak strength. After reaching the peak strength, the tangential shear stress suddenly decreased, indicating that a brittle shear failure mode occurred. Moreover, unlike the bending specimens, the stiffnesses of the tangential shear specimens in the same temperature group exhibited obvious discrepancies. The main reason for the discrepancies was the difference in the location of the shear plane. If the shear plane was formed in the early wood, the stiffness of the specimen was relatively low. In contrast, if the shear plane was in the late wood, the stiffness became relatively high. For the specimens at elevated temperatures, the curves generally exhibited similar shapes to those at room temperature (20 °C).

**Figure 8:**
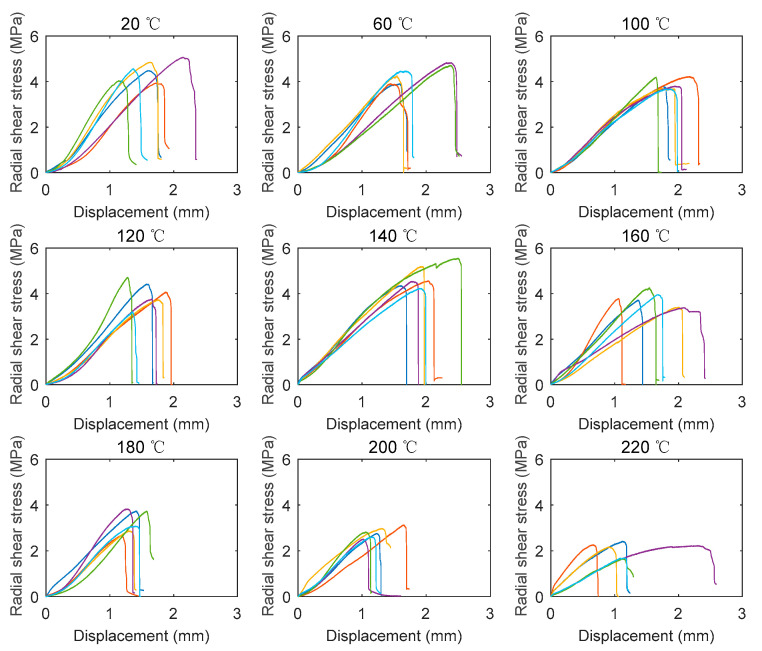
Radial shear stress–displacement at different elevated temperatures.

According to Equation (3), the radial shear stress–vertical displacement curves of all the specimens at different temperatures are shown in Figure 8. Apart from the initial loading stage, the radial shear stress increased approximately linearly up to the peak strength. Subsequently, the radial shear stress suddenly dropped, exhibiting the brittle shear behavior. The stiffness of the radial shear specimens in the same temperature group generally exhibited less discrepancy compared with that of the tangential shear specimens. Moreover, it seems that the temperature had a limited effect on the shape of the curve.

**Figure 9:**
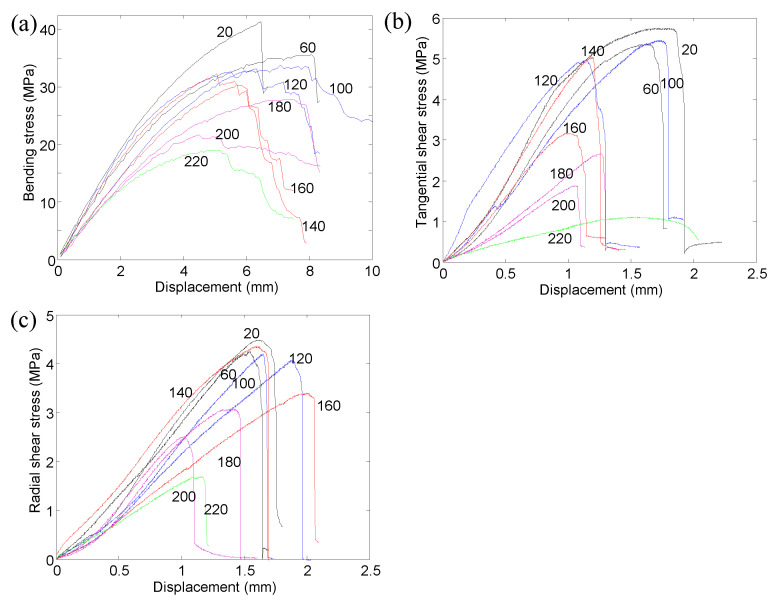
Typical stress–displacement curves: (**a**) three-point bending; (**b**) tangential shear; (**c**) radial shear.

Figure 9 depicts the typical stress–displacement curves of the specimens at different temperatures. It can be seen that the bending strength, tangential shear strength, and radial shear strength generally decreased with the increase in temperature. Moreover, the stiffnesses of these specimens generally reduced as the temperature increased.

### 3.3. Temperature-Dependent Strength

**Figure 10:**
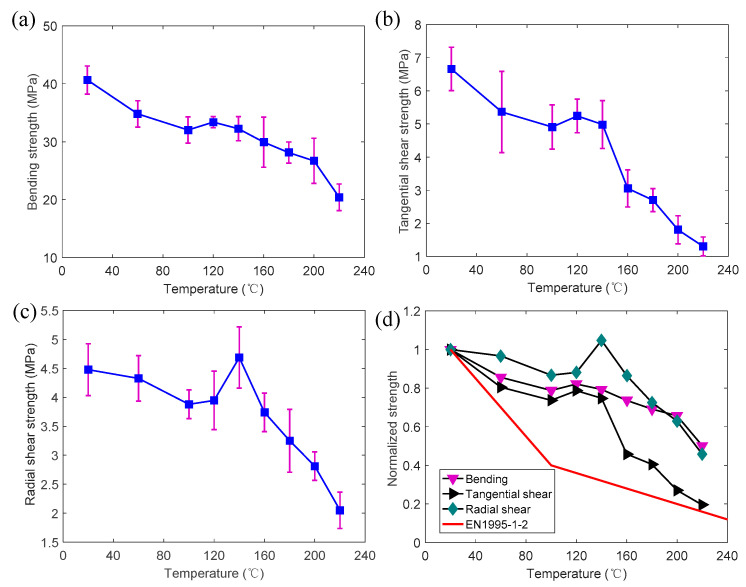
Temperature-dependent strength progressions with standard deviations (purple line): (**a**) bending; (**b**) tangential shear; (**c**) radial shear; (**d**) normalized value.

Figure 10a presents the bending strengths of the specimens at temperatures from 20 to 220 °C. The bending strength generally decreased with the increase in temperature. The bending strength decreased from approximately 40 MPa at 20 °C to 20 MPa at 220 °C. The average temperature-dependent normalized strengths (retention rates) of all the specimens are shown in Figure 10d. The retention rate of the bending strength significantly decreased from 1 at 20 °C to 0.79 at 100 °C. However, the retention rate slightly increased from 0.79 at 100 °C to 0.82 at 120 °C. Subsequently, the retention rate gradually reduced from 0.82 at 120 °C to 0.5 at 220 °C.

The effects of elevated temperatures on the tangential shear strength are shown in Figure 10b. The tangential shear strength decreased from 6.7 MPa at 20 °C to 4.9 MPa at 100 °C. Subsequently, the strength slightly increased to 5.2 MPa at 120 °C. Then the strength significantly reduced from 5.2 MPa at 120 °C to 1.3 MPa at 220 °C. It should be noted that the standard deviation of the tangential shear strength was greater than that of the bending strength. As shown in Figure 10d, the retention rate of the tangential shear strength dramatically reduced from 0.79 at 120 °C to 0.19 at 220 °C.

Figure 10c presents the radial shear strength at different temperatures. For the same temperature, the radial shear strength was significantly lower than the tangential shear strength. Moreover, the retention rate of the radial shear strength obviously increased from 0.87 at 100 °C to 1.05 at 140 °C. Subsequently, the retention rate of the radial shear strength reduced to 0.46 at 220 °C. The strength progressions for all the specimens can be classified into three stages according to the temperature range: (i) the significant reduction of strength from 20 to 100 °C; (ii) the improvement of strength from 100 to 120 (140) °C; and (iii) the reduction of strength from 120 (140) to 220 °C. This is consistent with the previous studies reported by Schaffer et al. [28], Irvine [29], Manríquez et al. [30], and Yue et al. [31]. The strength reduction in the first stage mainly came from the softening of the moist lignin and cellulose [29]. The increase in strength in the second stage mainly resulted from moisture evaporation [31]. Moreover, the strength reduction in the third stage came from the thermal degradation of the microstructures [28,31]. Gerhards [32] reviewed the studies on the immediate effects of moisture content and temperature on the bending and shear properties of defect-free wood. It was found that the mechanical properties (bending strength, MOE, and shear strength parallel-to-grain) were significantly affected by the moisture content and the temperature. The mechanical properties monotonously increased with the decrease in moisture content at the same temperature (20 °C). Moreover, the mechanical properties monotonously decreased with the increase in temperature at the same moisture content. However, in this study, the bending and shear properties did not exhibit a monotonous decrease as the temperature increased from 20 to 220 °C. It should be noted that the tests in this study were not conducted at a constant moisture content for different desired temperatures. As described above, the mechanical properties were affected by the moisture content and the temperature. In the first stage, the mechanical properties were mainly affected by the increase in temperature and softening of the wood, rather than the decrease in the moisture content. In the second stage, the improvement of the mechanical properties from the decrease in moisture content was higher than the degradation from the increase in temperature. In the third stage, since the moisture content was zero, the mechanical properties were only affected (degraded) as the temperature increased.

Figure 10d shows the design curve of EN 1995-1-2 for the shear strength at elevated temperatures. It can be found that the design curve is generally conservative to predict the tangential and radial shear strengths at elevated temperatures, especially within the temperature range between 100 and 140 °C. However, the retention rate of the tangential shear strength is 0.19 at 220 °C, which is slightly higher than the predicted value from EN 1995-1-2, indicating that the design curve may not be adopted to predict the tangential shear strength at temperatures higher than 220 °C.

### 3.4. Temperature-Dependent Modulus of Elasticity and Modulus of Rupture

**Figure 11:**
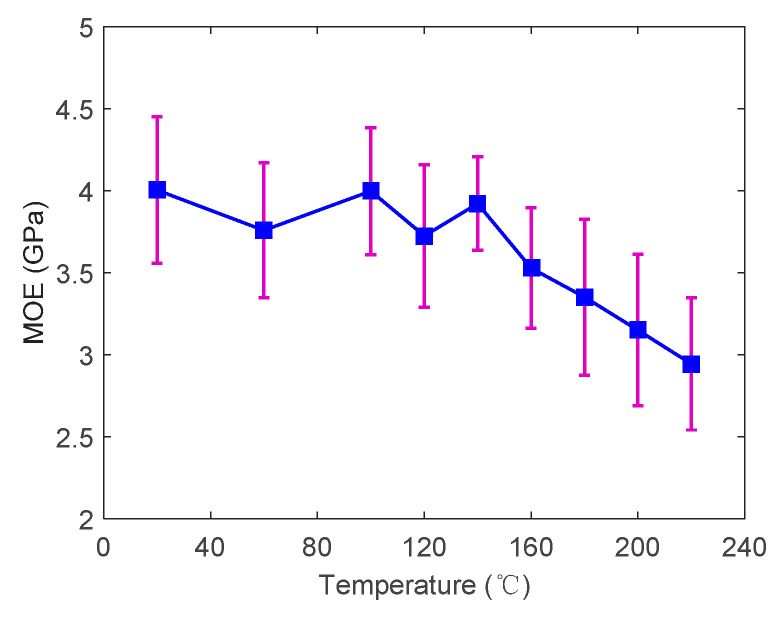
The modulus of elasticity with standard deviations (purple line) at elevated temperatures.

**Figure 12:**
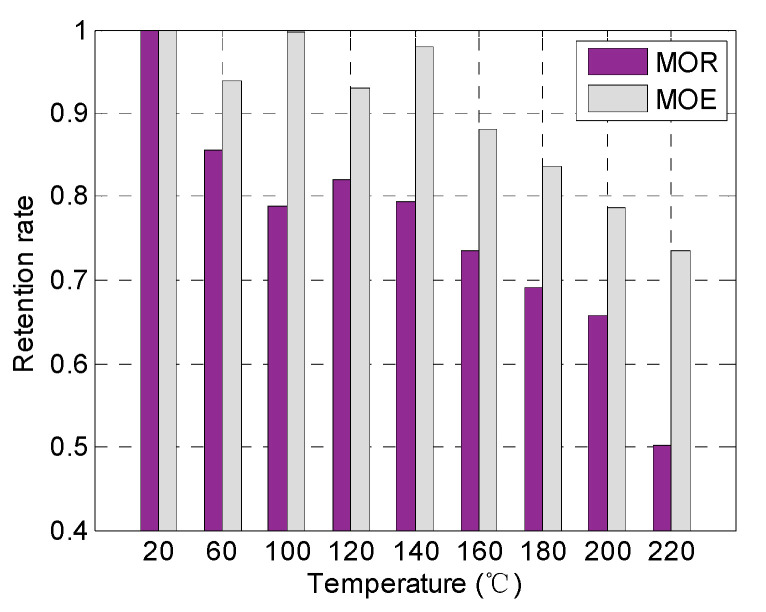
MOR and MOE degradation at elevated temperatures.

The MOE of the specimens at different temperatures is shown in Figure 11. Based on the average values and the corresponding standard deviations, no significant variation was found in the MOE from 20 to 140 °C. However, the MOE gradually decreased from 3.9 GPa at 140 °C to 2.9 GPa at 220 °C. Figure 12 presents the comparison between the retention rate of MOE and MOR at elevated temperatures. Under the same temperature, the retention rate of the MOE is significantly higher than that of the MOR. At 220 °C, the retention rate of the MOE is 1.5 times that of MOR. It should be noted that the MOE reflects the bending stiffness while the MOR reflects the bending strength. The MOR decreased significantly with the increase in temperature up to 100 °C. In contrast, the MOE started to reduce at a higher temperature (above 140 °C) where the softening of the wood material was more obvious. In other words, the decrease in MOE was delayed compared to the MOR.

### 3.5. Temperature-Dependent Deformation Capacity

**Figure 13:**
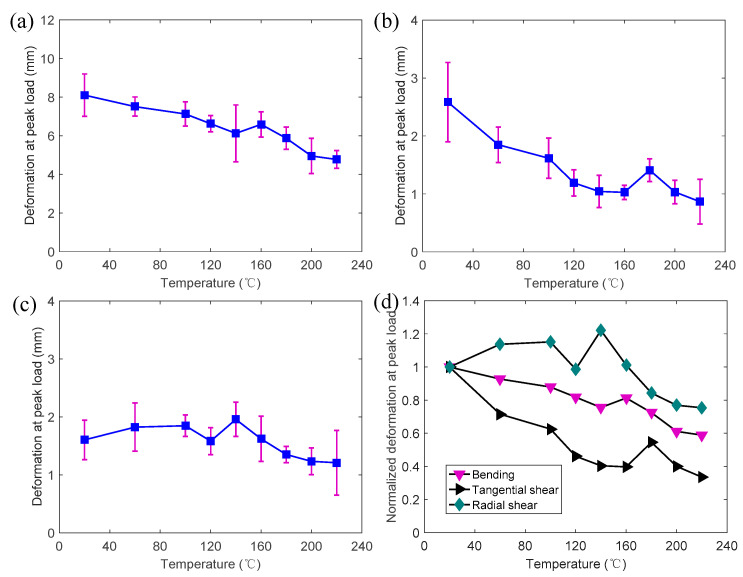
Temperature-dependent deformation at peak loads with standard deviations (purple line): (**a**) bending; (**b**) tangential shear; (**c**) radial shear; (**d**) normalized value.

Figure 13a presents the mid-span deformations of the bending specimens at the peak load under different temperatures. The deformation gradually decreased from 8.1 mm at 20 °C to 6.1 mm at 140 °C. Subsequently, the deformation increased slightly to 6.6 mm at 160 °C. Then the deformation reduced to 4.8 mm at 220 °C. As shown in Figure 13b, the deformation (vertical displacement of the cross-head) of the tangential shear specimens generally decreased from 2.6 mm at 20 °C to 1.0 mm at 160 °C. Then the deformation increased to 1.41 mm at 180 °C and then gradually decreased to 0.86 mm at 220 °C. Figure 13c presents the temperature-dependent deformations of the radial shear specimens at the peak load. The peak of the deformation may correlate to the peak of the strength. However, it can be observed that the peak of the deformation occurred at a higher temperature (140–180 °C) than the peak of the strength (120–140 °C). Therefore, the increase in the deformations mainly resulted from the glass transition (softening) of the polymer components, such as lignin and hemicellulose, from 120 to 180 °C. This is consistent with the studies reported by Beall and Eickner [33] and Irvine et al. [29], in which the hemicellulose and lignin were softened and partially pyrolyzed between 130 and 220 °C [31]. It can be seen that the deformations of the radial shear specimens and the tangential shear specimens were much lower than those of the bending specimens. Since the ductility of the wood commonly can be reflected by the deformation capacity, the ductility of the bending specimens was much higher than that of the shear specimens. Moreover, unlike the bending and tangential shear specimens, the temperatures had limited effects on the deformation of the radial shear specimens. Figure 13d presents the normalized average deformation for all specimens at different temperatures. Under the same temperature, the normalized deformation (retention rate) of the radial shear specimen was the largest while that of the tangential shear specimen was the smallest.

## 4. Conclusions

In this study, bending, tangential shear, and radial shear experiments were conducted on Chinese Paulownia wood at elevated temperatures between 20 to 220 °C. The effects of moisture content and temperature were invested at the same time. Based on the experimental results, the following conclusions can be drawn:

(1) Crushing–tensile rupture at the middle-span cross-section was the main failure mode of the bending specimen. Moreover, shear cracks were found in a few bending specimens. The tangential shear specimens exhibited straight cracks while radial shear specimens exhibited wave-form cracks on the bottom surfaces. Elevated temperatures seemed to have little effect on the failure modes.
(2) The bending and shear strengths were simultaneously affected by the moisture content and the elevated temperature. The strengths decreased with the increase in moisture content and temperature. In the low (20 to 100 °C) and high (140 to 220 °C) temperature ranges, the effect of the increase in temperature was dominant, whereas in the intermediate temperature range (100 to 140 °C) the effect of moisture content was dominant.
(3) The EN 1995-1-2 design curve for the shear strength of wood at elevated temperatures was conservative for both the tangential shear and radial shear specimens. However, the retention rate of the tangential shear strength was close to the predicted value from EN 1995-1-2 at 220 °C, indicating that the design curve may not be adopted to predict the tangential shear strengths at temperatures higher than 220 °C.
(4) The modulus of rupture (MOR) and the modulus of elasticity (MOE) generally decreased with the increasing temperature. Under the same temperature, the retention rate of the MOE was significantly higher than that of the MOR. At 220 °C, the retention rate of the MOE was 1.5 times that of MOR.
(5) The increase in the deformations at the peak load mainly resulted from the softening of the polymer components from 120 to 180 °C. The deformations of the radial shear specimens and the tangential shear specimens were much lower than those of the bending specimens. Moreover, most of the post-peak curves of the bending specimens exhibited pseudo-ductile characteristics due to the progressive damage. In contrast, the shear stress suddenly dropped after reaching the peak load, indicating that the shear specimens were much more brittle than the bending specimens.

## Figures and Tables

**Table 1:** Time required for heating the specimen to reach the desired temperature.

Specimen	Time Required to Reach the Desired Temperature (min)								
	20 °C	60 °C	100 °C	120 °C	140 °C	160 °C	180 °C	200 °C	220 °C
Bending	2.1	12.5	15.1	19.8	23.0	26.9	31.3	40.9	54.5
Tangential shear	2.5	15.9	28.4	32.1	35.4	37.5	44.4	52.3	64.8
Radial shear	2.5	16.1	30.3	34.2	36.8	38.6	44.9	53.1	65.5

## Data Availability

The data presented in this study are available on reasonable request from the corresponding author.
